# Psychophysiological Models to Identify and Monitor Elderly with a Cardiovascular Condition: The Added Value of Psychosocial Parameters to Routinely Applied Physiological Assessments

**DOI:** 10.3390/s20113240

**Published:** 2020-06-07

**Authors:** Victor Kallen, Jan Willem Marck, Jacqueline Stam, Amine Issa, Bruce Johnson, Nico van Meeteren

**Affiliations:** 1 The Netherlands Organization for applied Sciences (TNO), Anna van Buerenplein 1, 2595 DA The Hague, The Netherlands; jwmarck@gmail.com (J.W.M.); jacqueline.stam@tno.nl (J.S.); 2 The Physical Activity and Nutrition INfluences In ageing (PANINI) Consortium: School of Sport, Exercise and Rehabilitation Sciences, University of Birmingham, Edgbaston, Birmingham B15 2TT, UK; Meeteren@health-holland.com; 3 Department of Cardiovascular Medicine, Mayo Clinic, 200 First St. SW, Rochester, MN 55905, USA; issa.aemon@gmail.com (A.I.); johnson.bruce@mayo.edu (B.J.); 4 CAPHRI, Maastricht University, Minderbroedersberg 4–6, 6211 LK Maastricht, The Netherlands; 5 Top Sector Life Sciences & Health (Health~Holland), Wilhelmina van Pruisenweg 104, 2595 AN The Hague, The Netherlands

**Keywords:** elderly, cardiovascular, Heart Rate Variability, Heart Rate Recovery, respiratory, psychosocial functioning, prognostic model, depression, loneliness

## Abstract

The steadily growing elderly population calls for efficient, reliable and preferably ambulant health supervision. Since cardiovascular risk factors interact with psychosocial strain (e.g., depression), we investigated the potential contribution of psychosocial factors in discriminating generally healthy elderly from those with a cardiovascular condition, on and above routinely applied physiological assessments. Fifteen elderly (aged 60 to 88) with a cardiovascular diagnosis were compared to fifteen age and gender matched healthy peers. Six sequential standardized lab assessments were conducted (one every two weeks), including an autonomic test battery, a 6-min step test and questionnaires covering perceived psychological state and experiences over the previous two weeks. Specific combinations of physiological and psychological factors (most prominently symptoms of depression) effectively predicted (clinical) cardiovascular markers. Additionally, a highly significant prognostic model was found, including depressive symptoms, recently experienced negative events and social isolation. It appeared slightly superior in identifying elderly with or without a cardiovascular condition compared to a model that only included physiological parameters. Adding psychosocial parameters to cardiovascular assessments in elderly may consequently provide protocols that are significantly more efficient, relatively comfortable and technologically feasible in ambulant settings, without necessarily compromising prognostic accuracy.

## 1. Introduction

The steady increase of the elderly population (65 and older) encourages scientific efforts focused on healthy aging and elderly well-being [1]. Insights in the factors that either support or progressively undermine health and wellbeing may help to develop effective strategies to delay or even prevent disease onset and its functional consequences. A healthy condition in elderly should stimulate and support sustained independence (e.g., in living conditions), pro-social activities and societal participation. Chronic medical syndromes, like metabolic and/or cardiovascular conditions, are generally age related, and consequently constitute a severe challenge for such aims [2,3]. And it is nowadays well established that individual factors contribute significantly to their etiology, prognosis and outcomes. These factors include genetic vulnerabilities, lifestyle, life time physiological strain, psychological stress and contextual social and physical factors. For example, lower early life Social Economical Status (SES) is associated with significantly higher Risk Ratios (RR) for later life diabetes (RR = 1.23) and Cardiovascular Disease (CVD; RR = 1.30) [4]. Due to their chronic nature, these syndromes directly undermine the well-being and independence of those affected, and constitute a considerable strain on the health care system. Importantly, from psychosocial research we know that low SES is related to feelings of isolation [5], (negative) major life events and depression [6,7,8]. Such relations between aging, psychosocial constrains and CVD are clinically relevant and statistically interesting as they emphasize the validity of models and theories capturing the reciprocal and progressive influence of psychological and physiological strains on health outcomes, in particular in elderly [9].

Common diagnostic and prognostic methods primarily focus on physiological factors (e.g., in the establishment of CVD), typically assessed using extensive protocols executed in hospitals, whilst their accuracy and efficiency might potentially be strengthened by including psychosocial factors. This might enhance “efficiency”, as assessing psychosocial factors is generally cheaper, less intrusive and faster.

Previous scientific work clearly showed the potential relevance of prognostic models that include multisystem dynamics to predict health outcomes [10,11,12,13]. Models like the well-established allostasis concept [14] acknowledge a systemic, holistic and multifactorial perspective on health and (the development of) disease. It appeared that predictive values increase by integrating relevant (diagnostic) parameters from e.g., the (neuro)endocrine, physiological and psychological domains [13]. In this way, the impact of health threats, being either organic (viral, bacterial), psychophysiological (e.g., bereavement, exhaustion) or psychological (e.g., stress, feelings of loneliness and negative life events), and their combinations, can, at least theoretically, be captured. Scientific studies provide extensive support for the allostasis concept in the present context. For example, cognitive impairment has shown to strongly increase mortality among individuals suffering from immune diseases [15]; mood disorders are reported to be negatively associated with specific components of Heart Rate Variability (HRV), which are considered to be a prominent parameter of cardiovascular functioning and health [16]. Hence, the relevance of such multifactorial strategies and models, e.g., in the context of (healthy) aging, rapidly gains momentum [7,10].

In addition to the mentioned findings of the reciprocal effects of psychophysiological factors on health, evidence exists for social relationships to be directly related to physical and mental health and even mortality. Long-term detrimental health effects of negative social relations and social tensions appear to be comparable to those of smoking, obesity and lack of physical activity [17]. Other studies explicitly link mortality and health status with psychosocial factors as specific as social integration, social support, social disconnectedness and perceived isolation [18,19,20]. In relation to these findings, it has been convincingly shown that, although elderly seem to become better socially connected over the past decades (as compared to earlier birth cohorts), the aging effect on (feelings of) loneliness significantly outclasses this relatively small but statistically significant population effect. These findings emphasize that loneliness and (experienced) lack of self-efficacy are strongly related to aging and evident factors in health and well-being of elderly [21]. Both the direct relation with aging, and presumed (indirect) relations with cardiovascular parameters, again emphasize the potential relevance of psychosocial factors in health related prospects.

Consequently, in the present study we set out to investigate the potential of prognostic models combining physiological and psychosocial factors to discriminate between elderly with or without CVD. We hypothesize that including psychosocial factors (additionally to routinely applied diagnostic parameters for CVD) provides potentially simpler, thus more efficient, assessment methods, without necessarily compromising their prognostic accuracy.

## 2. Materials and Methods

### 2.1. Participants

Subjects included in this study were non-smoking, independently living elderly, aged between 60 and 88 years and able to provide informed consent. Subjects were assigned into one of two groups: 1) The CVD subjects: all diagnosed with chronic heart disease (CVD; N = 15) with either ischemic or non-ischemic cardiomyopathy for > 3 months, and no or slight functional limitations in physical activity (New York Health Association, NYHA, class I or II) [22]; 2) age/sex matched relatively healthy individuals (“reference group”; N = 15). General exclusion criteria were currently being hospitalized or having been hospitalized in the last 6 months (for any condition), and having a pacemaker. Participants in the CVD group were initially recruited through advertisements placed within a local independent living center and through actively contacting patients who had given prior permission for reviewing their medical record for research purposes. If inclusion criteria were met, independently living subjects in close proximity of the center were also included. Subjects for the reference group were recruited via an open invitation to the independently living center inhabitants with their medical status being verified with an intake interview. Consequently, the present study used a retrospective design. The study was conducted in accordance with the Declaration of Helsinki, and the protocol was approved by the Institutional ethical Review Board at Mayo Clinic, Rochester (IRB# 12-002896).

### 2.2. Procedure

The procedure consisted of two sequential stages: an intake and a series of 6 standardized lab assessments including an autonomic test battery, a 6-min step test and a selection of (self-report) psychological questionnaires. The assessments were conducted by two experienced staff members in a dedicated lab inside the independently living center. The intake questionnaires and the lab assessments were handed out and performed by trained lab technicians. Each lab visit lasted up to 75 min. There were no time limits set for the subjects during the lab assessments. Climate control remained constant at a comfortable level for the elderly.

Following the intake, covering an intake interview including medical status and (signing of) informed consent (copy available with the editor and/or the corresponding author), participants were asked to come to the lab every other week for six sequential assessments. Among other assessments, the biweekly set of questionnaires included the 12-item Short-Form version-2 (SF12v2) (assessing mental and physical Quality of Life; QoL): 12 items scored on a 5-point Likert scale (ranging from “all of the time” to “none of the time”); the PANAS Short Form (assessing positive and negative affect in the previous two weeks): 10 items scored on a 5-point Likert scale (ranging from “very slightly or not at all” to “a lot or often”); a listing of all positive and negative life events of the last two weeks; the Interpersonal Support Evaluation List (ISEL, experienced social support during the previous two weeks): 12 items scored on a 5-point Likert scale (ranging from “definitely false” to “definitely true”); a social integration questionnaire (measuring isolation, strength and extension of social network): 4 items scored on a 4-point Likert scale (ranging from “hardly ever or never” to “often”); and the Geriatric Depression Short Form (GDS-15, assessing depressive symptoms): 15 items, scored with “yes” or “no” [18,19,23,24,25].

Finishing the questionnaires and connecting the ECG leads typically required at least 20 min. This time was used as a resting period to physiologically recover from the walk to the laboratory, and to set a baseline for the physiological measures. As part of an autonomic test battery, subjects performed 5 min of paced breathing (5 s in, 5 s out). During the autonomic testing, a three lead ECG was recorded continuously using a Nexfin device [26]. The ECG recordings during paced breathing were used for HRV analysis.

Following, the 6-min step test was conducted, which consists of 2 min resting, 1 min stepping at 30 steps per minute (SPM), 1 min stepping at 60 SPM, 1 min stepping at 90 SPM and 1 min recovery. Following this exercise, the continued ECG recordings were used to determine Heart Rate Recovery (HRRec) [27]. During the exercise, multiple gas exchange variables were recorded to determine ventilatory efficiency and to calculate the Multi Variable Index (MVI) [28].

The MVI, originally developed to grade gas exchange deficiencies in CVD patients, was calculated using the following assessed variables: (1) quantification of breathing efficiency via ventilatory equivalents for carbon dioxide production (VE/VCO_2_); (2) Oxygen Uptake Efficiency Slope (OUES); (3) Oxygen Saturation (SaO_2_); (4) resting end-tidal carbon dioxide partial pressure (resting PetCO_2_); (5) PetCO_2_ change in response to exercise (ΔPetCO_2_); and (6) a standardized index for pulmonary capacitance (PCAP, being calculated by the oxygen pulse × PetCO_2_) [28].

### 2.3. Signal Processing & Analytics

From the ECG recordings, using the Librow matlab library, R peaks were determined [29]. Due to the frequent nature of various arrhythmias (PVC, atrial fibrillation, others) the normal RR intervals and the arrhythmic RR intervals were separated and analyzed independently. To detect and separate arrhythmic RR intervals, the squared difference of successive RR intervals was determined. The RR intervals with a squared successive difference larger than 0.015 (s^2^) were considered anomalous. The threshold of 0.015 (s^2^) was manually determined and provided reliable separation between arrhythmic periods and normal periods. If a file had less than 60% normal intervals, analysis on the normal intervals was discarded. For this reason, 20% of the original files were excluded.

#### 2.3.1. Time Domain Analysis

The data were visually inspected and, where needed, anomalous intervals were replaced by linear interpolation between the adjacent RR intervals. Time series of three or more sequential anomalous intervals were excluded from further analyses. Next, the standard deviation of all normal RR intervals (SDNN); and the Root Mean Squared Differences of Successive intervals (RMSSD) were calculated over the selected time series. Lower SDNN is generally associated with sympathetic activity and RMSSD is accepted as one of the most reliable parameters of vagal activity [30,31]. Associations between these parameters of heart rate variability and/or arrhythmias with psychosocial stressors have been reported extensively over the past decades, in particular in relation to cardiovascular risk factors and/or the types of psychosocial stress that are highly prevalent in elderly, like the passing away of a spouse [32,33]. Finally, Poincaré plots were used to determine Poincaré length (PC length) and width (PC width) for the non-arrhythmic sections.

#### 2.3.2. Frequency Domain Analysis

Applicable anomalous RR intervals were removed from the time series and resulting gaps were filled with values calculated by linear interpolation between the adjacent normal RR intervals. The RR time series were resampled at 10 samples per second. The time series were normalized subtracting the mean RR interval and were in this way detrended. The normalized resampled time series were analyzed using a fast Fourier transformation. From the power spectrum, two bands were used: low-frequency (LF: 0.04–0.15 Hz (ms^2^)) and high-frequency power (HF: 0.15–0.40 Hz (ms^2^)). These were normalized by dividing by the total power in the LF and HF bands resulting normalized low-frequency (LFN), related to sympathetic and vagal activity, and normalized high frequency (HFN), related to vagal activity [34,35]. The LF/HF ratio was regarded to be an index of sympathetic-vagal balance [30].

### 2.4. Statistical Analyses

A 3-step analytical strategy was applied. Firstly, to efficiently and reliably reduce the number of potential variables to be included in the eventual prognostic analytics, potential inter-domain relationships (e.g., between physiological and psychological variables) were investigated. To do so, the data over all six assessments were used as independent measurements in a correlation analysis. This eventually provided six times 23 observations (the data of 7 participants were incomplete): cumulative 138 datapoints for each variable, generating sufficient statistical power to be able to reliably calculate multiple correlations. Kolmogorov–Smirnov tests were applied to check for normality, which appeared to be applicable for all included variables. Consequently, we concluded that the parameters of interest met the assumptions for parametric analytical methods. The calculated correlations were evaluated on significance and over domains to correct for multiple testing. For example, only if all HRV parameters correlated with a psychological or social outcome, the results were used for further analyses. Single or relatively random correlations were considered unreliable (even when Bonferroni corrected) and prone for chance capitalization and were consequently discarded.

Secondly, the identified and validated relations between psychosocial and physiological parameters were implemented in multiple regression analyses. Three backward regression analyses were done to calculate the best fitting regression models combining physiological and psychological factors to predict the relevant CVD risk indicators, being (sequentially) MVI, VEVCO_2_Slope and PO_2_P. Thereafter, these analyses were repeated for both groups separately, to further explore variability of cardiovascular risk markers in both groups.

Thirdly, to identify the potential prognostic accuracy of the hypothesized multi-modular models, multiple stepwise logistic regression analyses were applied with group membership as a dependent variable. Firstly, a model was constructed that incorporated routinely assessed physiological factors only (physiological model). Following, the preselected psychosocial parameters (based on the correlation analyses) were added to identify the strongest composite (physiological and psychosocial) model: psychophysiological model. Finally, to be able to compare the prognostic accuracy, a Receiver-Operator Curve (ROC) was designed and the Area Under the Curve (AUC) was calculated for these two (physiological and psychophysiological) models.

Results with *p*-values < 0.05 were considered to represent significant findings and are consequently reported. Regression models with an R^2^ > 0.30 were considered to be potentially relevant. Consequently, findings with R^2^ < 0.30 are occasionally reported, although not considered accurate enough for prognostic means.

## 3. Results

### 3.1. Sample Statistics

Four recruited individuals dropped out during the intake procedure (2 reference group; 2 CVD) due to personal or medical reasons, or to the demanding nature of the study. Two individuals ceased participation later (1 reference, 1 CVD), one due to the development of an overt clinical depression and one for personal medical reasons. One subject from the reference group entered the study 30 days after the initial starting date and thus misses one out of six data points. The presented results were consequently based on 23 participants who finished the study. No significant group differences were found in age, male/female ratio, or BMI. Combined with the rather homogeneous networks and living environments used for recruitment, the latter finding suggested a generally similar lifestyle over all participants. Data from medical records on Left Ventricular Ejection Fraction (LVEF) and diagnosed arrhythmias confirmed accurate group assignment (both significantly worse in the CVD group; see Table 1).

### 3.2. Variable Reduction and Selection

The first analytical step was to calculate a correlation matrix (including all 6 assessments as independent observations) to map significant interactions between specific psychosocial and physiological variables. As expected, the intradomain correlations were generally high (e.g., self-reported negative life events and mental QoL: r = −0.74; depression and social isolation: r = 0.84; RMSSD and SDNN: r = 0.69, all *p*’s < 0.05). Interdomain (physiological, psychological and social) correlations appeared to cluster on some CVD markers (e.g., MVI and social support: r = 0.34, *p* < 0.05), although this was clearer on emotional functioning and Heart Rate Variability components, as presented in Table 2.

Elaborating on the statistics in Table 2 (representing only singular relations), two additional regression analyses showed that the combination of age, depression, RMSSD and HRRec best predicted mental QoL (F(5, 51) = 5.14, *p* = 0.001, R^2^ = 0.36); and the combination of depression, RMSSD, SDNN and HRRec appeared to be most predictive for physical Qol, though with only a mediocre explained variance (R^2^ = 0.23). Additionally, experienced negative life events during the previous two weeks appeared to be associated with age, depression, isolation and mental QoL (F(4, 37) = 13.05, *p* < 0.0005). The explained variances increased with the sequential addition of cardiovascular factors: arrhythmia parameters (RMSSD and SDNN) appeared to be the most prominent (F(6, 37) = 9.74, *p* < 0.0005, R^2^ = 0.65). However, positive life events were best predicted by models incorporating social factors and global parameters of HRV (RMSDD and SDNN); e.g., combining isolation, mental Qol, social support, SDNN, HF/LF ratio and HF (F(6, 37) = 4.59, *p* = 0.002, R^2^ = 0.47).

### 3.3. Psychosocial Variables in Relation to CVD Risk Indicators

The Multi Variable Index (MVI), or minute ventilation-to-carbon dioxide output slope (VEVCO_2_Slope), and peak oxygen pulse (PO_2_P) during the 6-min step test are widely used in clinical practice to evaluate symptom severity of patients suffering from chronic heart failure. The results of the previous correlational analyses were plugged into three backward regression analyses aimed at identifying the interactive relationship of physiological and psychosocial factors with these established CVD risk indicators. This provided multiple significant prediction models.

Using MVI as dependent variable, four significant models appeared. The strongest model (F(6, 54) = 3.86, *p* = 0.003, R^2^ = 0.33) included age, SDNN, depression and RMSSD as independent variables. Another model with even less independent variables and comparable predictive power (F(3, 54) = 5.85, *p* = 0.002, R^2^ = 0.26) incorporated age, depression and RMSSD.

Two significant models predicted VEVCO_2_Slope, neither however with convincing statistical strength. The smallest model (R^2^ = 0.12) included RMSSD and depression (F(2, 57) = 3.82, *p* = 0.028).

Five significant models could be formulated with regards to PO_2_P (dependent variable), ranging in explained variances (R^2^) from 0.43 to 0.46. The model of preference (with the least independent variables with comparable explained variance) included age, gender, depression and RMSSD (R^2^ = 0.46; F(5, 48) = 7.3, *p* < 0.0005). Two models incorporating age, gender and RMSSD (excluding social emotional factors) appeared to be slightly less powerful (R^2^ = 0.43).

When the same statistical exercise was conducted in both samples separately, in the reference group only minor nuances were found in the predictive models, and for MVI only. The best model included age, depression, HRRec (previously excluded), SDNN and RMSSD as independent variables (F(5, 31) = 7.44, *p* < 0.0005, R^2^ = 0.59).

Interestingly, in the CVD group two additional significant predictive models were found for MVI, and a third on trend level. The first model incorporated only depression as independent factor (F(1, 19) = 5.14, *p* = 0.036, R^2^ = 0.22), whereas the second incorporated depression and SDNN (F(2, 19) = 3.82, *p* = 0.043, R^2^ = 0.31). In the third model age and RMSSD were added as significant predictive factors (F(3, 19) = 3.19), *p* = 0.052, R^2^ = 0.37).

### 3.4. Prognostic Models Combining Physiological and Psychosocial Factors: Predicting High CVD Risk

Following, by using stepwise logistic regression analyses, multiple significant models were identified classifying reference and CVD participants. Compared to a model incorporating commonly assessed physiological markers only, the most accurate model appeared to include depressive symptoms, negative events and experienced social isolation (see Table 3).

## 4. Discussion

The present explorative observational study set out to investigate whether psychosocial factors may add additional predictive and prognostic value up and above the commonly used clinical (generally physiological) indicators of heightened cardiovascular risk in elderly. After being assessed on psychosocial functioning, 23 elderly (aged 60 to 88 years) were tested every other week for 3 months. Cardiovascular, respiratory and psychosocial parameters were evaluated along with QoL and recent positive and negative events. This provided an extensive database which was used to construct psychophysiological regression models to predict parameters of cardiovascular risk status and prognostic models combining physiological and psychosocial variables aimed at differentiating between elderly either or not at risk for the development of (chronic) cardiovascular disease.

With the aim of data reduction, and based on the cumulative data of the six sequential assessments, a correlation matrix identified the clusters of relevant, highly correlated variables. Not surprisingly, correlations appeared to be high within domains (e.g., emotional functioning, cardiovascular variables or clinical markers). Additional clusters of correlations were identified, generally confirming established relations, for example between specific parameters of HRV, mourning, rumination and/or symptoms of depression (see Table 2) [32,36]. Further analyses confirmed the relations between the diverse cardiovascular variables (SDNN, HRRec and RMSSD) with the relevant psychosocial factors like experienced social support and QoL. For example, depression scores combined with HRV and HRRec outcomes appeared to present stronger relations with both physical and mental QoL. With regards to mental QoL, it is interesting to notice that physiological factors (generally HRV components) play a prominent role, generally associated with mood (e.g., depressive states). This suggests that with better cardiovascular condition, depressive tendencies may decrease and QoL might increase, providing opportunities for unconventional, lifestyle based interventions, like well-tailored and evidence based individual exercise and nutrition programs.

Based on the previous steps, regression analyses were applied to identify the most accurate combinations of factors to predict clinical indicators of CVD (MVI, VEVCO_2_Slope and PO_2_P). These analyses provided multiple significant models for MVI and PO_2_P. Depression scores appeared to be a prominent factor providing slightly superior statistics over models without psychosocial factors. However, the preselected set of independent variables did not provide satisfying models for VEVCO_2_Slope, a variable potentially more closely related to physiological and cardiovascular disturbances. Interestingly, in the healthy elderly, HRRec appeared to be a dominant factor in both QoL and in relation to clinical indicators. This finding is in accordance with previous findings identifying HRRec as a representative parameter of fitness in generally healthy individuals [37].

Nevertheless, using stepwise backward regression analyses, most presented models combine physiological and psychosocial factors. The few models that only comprised physiological factors (exclusively applying to PO_2_P) appeared to be significantly inferior. As a consequence, we may conclude that adding psychosocial factors to models predicting CVD risk in elderly may very likely be a useful contribution, not least to increase predictive power. Specifically, adding depression scores appears to provide a statistical surplus in modeling cardiovascular risk outcomes (chronic heart failure/CVD) on and above well-known cardiovascular parameters. When both CVD and reference samples were analyzed separately, in the CVD group depression scores gained even more statistical prominence. This suggests that in individuals experiencing symptoms of chronic heart disease, even if they are mild, depressive symptoms may become an increasingly relevant indicator of the presently used health outcomes. However, more research in more robust and heterogeneous samples (e.g., considering smoking, obesity and lack of physical activity) is necessary to further unravel the (potential) contribution of symptoms of depression in the prognosis of cardiovascular risk in the elderly population.

### 4.1. Investigating the Contribution of Psychosocial Factors in Prognostic Accuracy

Using the above mentioned steps for data reduction and variable selection, a physiological model was established to define prognostic accuracy, based on established cardiovascular and clinical markers for CVD (including MVI, Age, RMSSD and HRRec), which appeared to be superior to most models including psychological factors in discriminating between high and low risk elderly. However, a psychophysiological model including a wide variety of psychosocial and cardiovascular parameters appeared to be the most accurate (see Table 3). This last finding strongly suggests that factors like recent negative life events, (feelings of) social isolation and symptoms of depression contribute significantly to the prognosis of elderly with a cardiovascular risk status.

### 4.2. Limitations of the Study

Unfortunately, two elderly with significant initial depression scores and a notable negative outlook on life prematurely resigned from the study. Given their state of mind, this was not a surprise, although this significantly decreased the distribution within the sample specifically in the negative direction. Consequently, stronger, and potentially more valid and representative effects may have been observed than those reported here.

Secondly, the present sample was rather small. Consequently, some methodological concessions had to be made, primarily with respect to the applied statistical strategy and methods. For example, statistical power was insufficient to engage in more sophisticated repeated measurement analytical designs. This, of course, limits the interpretation and generalizability of the presented results. Additionally, all participants were non-smokers, possibly biasing the found results to some extent as compared to the general population. On the other hand, the participating elderly, both from the participating institute and those coming from other locations appeared to be remarkably compliant and motivated to participate in six consecutive assessment rounds in a period of 3 months. This provided a considerable body of data, resulting in outcomes that are generally in line with previously reported findings (e.g., in relation to depression and HRV, or the applicability of HRRec to quantify fitness, even in populations like the present one) [27].

In relation to that last point, it must be noted that several parameters of HRV applied here may actually reflect overlapping sources of variance. The appropriate interpretation of (short time series of) SDNN, for example, is yet under debate [38]. In addition, although RMSSD is suggested to be closely associated with HF HRV [30], its contribution over distinct frequency ranges might not be uniform, causing at least some ambiguity in its interpretation. However, it is remarkable to notice that these more ‘global’ and less specific parameters of HRV showed the strongest association with the included physiological risk factors and most profound contribution to the ‘prognostic’ logistic models. This finding does entail a major practical advantage: the assessment of SDNN and RMSSD requires less complex proceedings, sensors and data processing software than the assessment of frequency derived parameters (i.e., LF or HF HRV). This would make the assessment of relevant parameters in an ambulant setting realistic and achievable.

Lastly, although the present findings underline the prognostic potency of combining cardiovascular, physiological and psychosocial factors in adequately identifying elderly at risk for CVD, to effectively implement such prognostic strategies in clinical practice might yet be a challenge in itself. It requires the combination of expertise, skills and technologies from quite specific domains in, more or less standardized, assessment batteries. Nevertheless, in the context of personalized medicine, and supported by developments in sensor and analytical technologies, models like the ones outlined here might be of significant importance for clinical decision making in elderly in the near future.

### 4.3. Clinical and Technological Implications

The use of Artificial Intelligence to process ECG tracings into valid and reliable health risk indicators (for example in relation to potassium changes or ejection fraction) progresses steadily into clinical practice and routine. This development is paralleled by the unpreceded current developments in sensory technology. An important example of the latter is the assessment of cardiovascular and psychophysiological parameters via smartphones, a development that seems to quickly mature into reliable and valid assessment methods [39]. The combination of smart sensor devices with accurate and valid algorithms (potentially of the signature outlined in the present study) creates novel opportunities in effective large scale monitoring of high risk populations. In this way, monitoring elderly in their own living environment might help to significantly reduce the frequency of visits to medical institutes for relatively routine assessments. As there are quite some studies covering either the physiological or the psychological factors associated with (cardiovascular) risk status, efforts to merge these factors in a meaningful way may be a useful step forward in efficiently and reliably tracking health status.

### 4.4. Conclusions

In the present study, we set out to explore the potential additional contribution of psychosocial factors in identifying elderly at risk for cardiovascular disease on-and-above routinely used cardiovascular variables and clinical parameters. With this aim, the present sample of elderly provided prognostic models with comparable and sometimes even superior accuracy over models incorporating established cardiovascular or clinical markers only. Apart from the found contribution in prognostic accuracy, an additional advantage might be that assessing the relevant psychosocial factors (e.g., depression, quality and quantity of social relations and recent negative events) can be done rather efficiently, for example by means of mobile technology. Consequently, extending diagnostic routines with assessments aimed at these factors might be considered useful. Nevertheless, future studies are necessary to shed more light on the presently reported relations; to further develop the here proposed prognostic strategies; and to tackle associated technological assessment challenges.

## Figures and Tables

**Table 1:** Subject characterization and relevant medical indicators (LVEF, % arrhythmias). Means and standard deviations (SD) are reported unless otherwise indicated.

	Reference	CVD	Total
N	14	9	23
Age (years)	77 ± 7	72 ± 10	75 ± 8
M/F ratio	10/4	7/2	17/6
BMI	25 ± 2	27 ± 3	26 ± 2
LVEF	>60%	43% ± 8	-
% Diagnosed with arrhythmias	7% (1 of 14)	78% (7 of 9)	35% (8 of 23)

Note: BMI = Body Mass Index; LVEF = Left Ventricular Ejection Fraction.

**Table 2:** Clustered Pearson’s correlation coefficients of psychosocial parameters with Heart Rate (Variability) parameters. Only significant (*p* < 0.05) correlations are reported.

	HR	SDNN	RMSSD	PC Width	HF/LF Ratio	HF	LF
**Depression**		−0.44	−0.36	0.47			
**Negative events**	0.19	−0.31	−0.43	0.39	−0.16	−0.16	0.20
**Isolation**	−0.15	−0.21	−0.25	0.40		−0.15	0.18
**Positive events**	0.54		−0.20	−0.28	−0.38	−0.43	0.32
**Social support**		0.22	0.31				
**Mental QoL**	−0.15	0.26	0.44	−0.33	0.15	0.16	−0.20
**Physical QoL**	0.37	0.35		−0.16			

Note: HR = Heart Rate; SDNN = Standard Deviation of NN (corrected RR-top) intervals; RMSSD = Root Mean Squared Successive Difference of sequential RR intervals; PC = Poincaré; HF = High Frequency (0.15–0.40 Hz); LF = Low Frequency (0.04–0.15 Hz).

**Table 3:** Test statistics of the physiological reference model for Cardiovascular Disease (CVD) vs. reference group, as compared to an extended psychophysiological model.

Model	Parameters	Chi^2^	Df	*p*	% TP	AUC
**Physiological**	MVI, Age, RMSSD, HRREC	21.11	5	0.001	83%	0.78
**Psychophysiological**	VEVCO_2_Slope, HRRec, MVI, Depression, Negative events, Isolation, SDNN, RMSSD	28.81	8	<0.0005	86%	0.83

Note: % TP = Percentage True Positives (correctly classified CVD patients).

## Data Availability

Deidentified participant data are available upon reasonable request via the corresponding author (victor.kallen@tno.nl).

## References

[1] Whittaker A.C., Delledonne M., Finni T., Garagnani P., Greig C., Kallen V., Kokko K., Lord J., Maier A.B., Meskers C.G.M. (2018). Physical Activity and Nutrition INfluences In ageing (PANINI): Consortium mission statement. Aging Clin. Exp. Res..

[2] Katon W., Lyles C.R., Parker M.M., Karter A.J., Huang E.S., Whitmer R.A. (2012). Association of depression with increased risk of dementia in patients with type 2 diabetes: The diabetes and aging study. Arch. Gen. Psychiatry.

[3] Lakatta E.G. (2002). Age-associated cardiovascular changes in health: Impact on cardiovascular disease in older persons. Heart Fail. Rev..

[4] Nandi A., Glymour M.M., Kawachi I., VanderWeele T.J. (2012). Using marginal structural models to estimate the direct effect of adverse childhood social conditions on onset of heart disease, diabetes, and stroke. Epidemiology.

[5] Beutel M.E., Klein E.M., Brähler E., Reiner I., Jünger C., Michal M., Wiltink J., Wild P.S., Münzel T., Lackner K.J. (2017). Loneliness in the general population: Prevalence, determinants and relations to mental health. BMC Psychiatry.

[6] Logan J.G., Barksdale D.J. (2008). Allostasis and allostatic load: Expanding the discourse on stress and cardiovascular disease. J. Clin. Nurs..

[7] Stephens C., Alpass F., Towers A., Stevenson B. (2011). The effects of types of social networks, perceived social support, and loneliness on the health of older people: Accounting for the social context. J. Aging Health.

[8] Lorant V., Deliège D., Eaton W., Robert A., Philippot P., Ansseau M. (2003). Socioeconomic inequalities in depression: A meta-analysis. Am. J. Epidemiol..

[9] Frasure-Smith N., Lespérance F., Habra M., Talajic M., Khairy P., Dorian P., Roy D. (2009). Elevated depression symptoms predict long-term cardiovascular mortality in patients with atrial fibrillation and heart failure. Circulation.

[10] Seeman T.E., McEwen B.S., Rowe J.W., Singer B.H. (2001). Allostatic load as a marker of cumulative biological risk: MacArthur studies of successful aging. Proc. Natl. Acad. Sci. USA.

[11] Karlamangla A.S., Singer B.H., McEwen B.S., Rowe J.W., Seeman T.E. (2002). Allostatic load as a predictor of functional decline: MacArthur studies of successful aging. J. Clin. Epidemiol..

[12] Juster R.P., Bizik G., Picard M., Arsenault-Lapierre G., Sindi S., Trepanier L., Marin M.F., Wan N., Sekerovic Z., Lord C. (2011). A transdisciplinary perspective of chronic stress in relation to psychopathology throughout life span development. Dev. Psychopathol..

[13] Juster R.P., McEwen B.S., Lupien S.J. (2010). Allostatic load biomarkers of chronic stress and impact on health and cognition. Neurosci. Biobehav. Rev..

[14] Mcewen B.S., Gianaros P.J. (2010). Central role of the brain in stress and adaptation: Links to socioeconomic status, health, and disease. Ann. N. Y. Acad. Sci..

[15] Wikby A., Ferguson F., Forsey R., Thompson J., Strindhall J., Löfgren S., Nilsson B.O., Ernerudh J., Pawelec G., Johansson B. (2005). An immune risk phenotype, cognitive impairment, and survival in very late life: Impact of allostatic load in Swedish octogenarian and nonagenarian humans. J. Gerontol. Ser. A Biol. Sci. Med. Sci..

[16] Shinba T., Kariya N., Matsui Y., Ozawa N., Matsuda Y., Yamamoto K.I. (2008). Decrease in heart rate variability response to task is related to anxiety and depressiveness in normal subjects. Psychiatry Clin. Neurosci..

[17] Holt-Lunstad J., Smith T.B., Layton J.B. (2010). Social relationships and mortality risk: A meta-analytic review. PLoS Med..

[18] Cornwell E.Y., Waite L.J. (2009). Social disconnectedness, perceived isolation, and health among older adults. J. Health Soc. Behav..

[19] Gallant M.P. (2003). The influence of social support on chronic illness self-management: A review and directions for research. Health Educ. Behav..

[20] Cohen S. (2004). Social relationships and health. Am. Psychol..

[21] Suanet B., van Tilburg T.G. (2019). Loneliness declines across birth cohorts: The impact of mastery and self-efficacy. Psychol. Aging.

[22] Goldman L., Hashimoto B., Cook E.F., Loscalzo A. (1981). Comparative reproducibility and validity of systems for assessing cardiovascular functional class: Advantages of a new specific activity scale. Circulation.

[23] Cheak-Zamora N.C., Wyrwich K.W., McBride T.D. (2009). Reliability and validity of the SF-12v2 in the medical expenditure panel survey. Qual. Life Res..

[24] Thompson E.R. (2007). Development and validation of an internationally reliable short-form of the Positive and Negative Affect Schedule (PANAS). J. Cross. Cult. Psychol..

[25] Sheikh J.I., Yesavage J.A. (1986). 9/geriatric depression scale (Gds) recent evidence and development of a shorter version. Clin. Gerontol..

[26] Eeftinck Schattenkerk D.W., Van Lieshout J.J., Van Den Meiracker A.H., Wesseling K.R., Blanc S., Wieling W., Van Montfrans G.A., Settels J.J., Wesseling K.H., Westerhof B.E. (2009). Nexfin noninvasive continuous blood pressure validated against Riva-Rocci/Korotkoff. Am. J. Hypertens..

[27] Snoek J.A., Van Berkel S., Van Meeteren N., Backx F.J.G., Daanen H.A.M. (2013). Effect of aerobic training on heart rate recovery in patients with established heart disease; a systematic review. PLoS ONE.

[28] Kim C.H., Anderson S., MacCarter D., Johnson B. (2012). A multivariable index for grading exercise gas exchange severity in patients with pulmonary arterial hypertension and heart failure. Pulm. Med..

[29] Librow. www.librow.com.

[30] Goedhart A.D., Van Der Sluis S., Houtveen J.H., Willemsen G., De Geus E.J.C. (2007). Comparison of time and frequency domain measures of RSA in ambulatory recordings. Psychophysiology.

[31] Hemingway H., Malik M., Marmot M. (2001). Social and psychosocial influences on sudden cardiac death, ventricular arrhythmia and cardiac autonomic function. Eur. Heart J..

[32] Fagundes C.P., Murdock K.W., LeRoy A., Baameur F., Thayer J.F., Heijnen C. (2018). Spousal bereavement is associated with more pronounced ex vivo cytokine production and lower heart rate variability: Mechanisms underlying cardiovascular risk?. Psychoneuroendocrinology.

[33] Thayer J.F., Yamamoto S.S., Brosschot J.F. (2010). The relationship of autonomic imbalance, heart rate variability and cardiovascular disease risk factors. Int. J. Cardiol..

[34] Malliani A., Lombardi F., Pagani M. (1994). Power spectrum analysis of heart rate variability: A tool to explore neural regulatory mechanisms. Br. Heart J..

[35] Ori Z., Monir G., Weiss J., Sayhouni X., Singer D.H. (1992). Heart rate variability: Frequency domain analysis. Cardiol. Clin..

[36] Carnevali L., Thayer J.F., Brosschot J.F., Ottaviani C. (2018). Heart rate variability mediates the link between rumination and depressive symptoms: A longitudinal study. Int. J. Psychophysiol..

[37] Daanen H.A.M., Lamberts R.P., Kallen V.L., Jin A., Van Meeteren N.L.U. (2012). A systematic review on heart-rate recovery to monitor changes in training status in athletes. Int. J. Sports Physiol. Perform..

[38] Shaffer F., Ginsberg J.P. (2017). An Overview of Heart Rate Variability Metrics and Norms. Front. Public Health.

[39] Plews D.J., Scott B., Altini M., Wood M., Kilding A.E., Laursen P.B. (2017). Comparison of heart-rate-variability recording with smartphone photoplethysmography, polar H7 chest strap, and electrocardiography. Int. J. Sports Physiol. Perform..

